# Improvement of montmorillonite adsorption capacity for lead ions by modifying with hexadecyl trimethyl ammonium chloride: Characterization, modelling and optimization studies

**DOI:** 10.1016/j.mex.2019.09.032

**Published:** 2019-09-25

**Authors:** Mohamadreza Massoudinejad, Syed Mohsen Mohseni, Mansour Ghaderpoori, Maryam Sarkhosh, Soleyman Sahebi

**Affiliations:** a Member of Safety Promotion and Injury Prevention Research Center, And Professor of Environmental Health Engineering, School of Public Health, Shahid Beheshti University of Medical Sciences, Tehran, Iran; b Department of Environmental Health Engineering, School of Public Health, Shahid Beheshti University of Medical Science, Tehran, Iran; c Department of Environmental Health Engineering, School of Health and Nutrition, Lorestan University of Medical Sciences, Khorramabad, Iran; d Nutritional Health Research Center, Lorestan University of Medical Sciences, Khorramabad, Iran; e Department of Environmental Health Engineering, Social Determinants of Health Research Center, Mashhad University of Medical Sciences, Mashhad, Iran; f Department for Management of Science and Technology Development, Ton Duc Thang University, Ho Chi Minh City, Viet Nam; g Faculty of Environment and Labor Safety, Ton Duc Thang University, Ho Chi Minh City, Viet Nam

**Keywords:** Montmorillonite modified with hexadecyl trimethyl ammonium chloride surfactant (N-HTAC) was applied as a new adsorbent for the adsorption of Pb (II) ions, Nano clay, Hexadecyl trimethyl ammonium chloride, Pb (II), Modeling, Response surface methodology, Optimization

## Abstract

Heavy metal pollutants, particularly Pb are considered as critical contaminants causing harmful health risks for a human. In this study, montmorillonite modified with hexadecyl trimethyl ammonium chloride surfactant (N-HTAC) was applied as a new adsorbent for the Pb^+2^ adsorption from aqueous solutions. The N-HTAC was characterized by the scanning electron microscopy, x-ray diffraction, energy-dispersive x-ray spectroscopy, and Brunauer-Emmett-Teller. The central composite design using R software was chosen for modelling the effect of operating parameters. Based on the findings obtained from the analysis of variance, reduced full second-order model with multiple R^2^, 0.94, adjusted R^2^, 0.93, and LoF, 0.96, was represented satisfactory adjustment with experimental data. The Solver “add-ins” was employed to gain the optimum conditions for the modelling. The optimum operating points giving the maximum Pb^+2^ removal (99.99%), were found to be initial Pb^+2^ concentration: 0.1 mg L^1−^ adsorbent dosage: 4.33 g L^-1^, HTAC dosage: 4.19 g L^-1^, pH: 7.13, temperature: 28.06^ºC^, and the reaction time: 103.4 min. The findings of the study showed that by enhancing and improving natural adsorbents, a significant amount of environmental pollutants can be eliminated.

• In this study, a new modified adsorbent (N-HTAC) was used to remove lead ions.
• The results of this study showed that the N-HTAC used has high efficiency (99.99%) in the removal of lead.
• The results of this study and the data obtained can be used to supplement the information on the removal of contaminants with adsorbents.

## Graphical abstract

**Figure:**
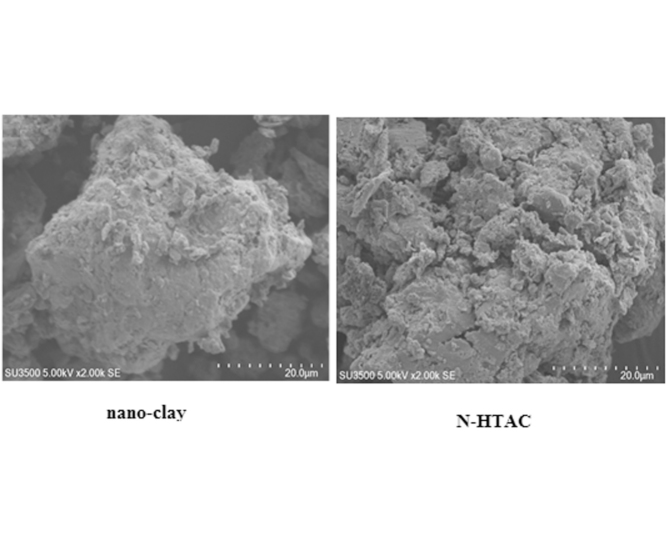

**Specifications Table**

**Table:** 

Subject area:	Environmental Science
More specific subject area:	Water and wastewater treatment
Method name:	montmorillonite modified with hexadecyl trimethyl ammonium chloride surfactant (N-HTAC) was applied as a new adsorbent for the adsorption of Pb (II) ions
Name and reference of the original method:	M. Massoudinejad, M. Ghaderpoori, A. Shahsavani, A. Jafari, B. Kamarehie, A. Ghaderpoury, M.M. Amini, Ethylenediamine-functionalized cubic ZIF-8 for arsenic adsorption from aqueous solution: Modeling, isotherms, kinetics and thermodynamics, Journal of Molecular Liquids, 255 (2018) 263-268
Resource availability:	The data are available with this article

## Method details

According to the *World Health Organization (WHO)* and the *United State Environmental Protection Agency* (*EPA*), cadmium (Cd), lead (Pb), and mercury (Hg) are toxic and extremely dangerous even at trace levels. Since these heavy metals have a cumulative property in human and animal soft tissues [1]. *WHO* and *USEPA* have set the maximum permissible drinking water limits of 0.05 mg L^−1^ and 0.015 mg L^−1^ for Pb, respectively [2]. Textile, leather, paper and pigments, steel fabrications, glass, electroplating, mining operations, and photographic materials are the main sources of Pb into the environment [3]. If the heavy metals are not properly treated then can cause a serious problem for living organisms and public health [4]. In the environment, Pb typically exists in the state of (II) and (IV) where Pb^2+^ is a common industrial pollutant posing serious ecosystem threats [5]. Speciation of Pb compound solely relies on pH, dissolved oxygen, concentration of other organic, and an inorganic compound. So far, various methods have been employed for removing Pb from water sources. These methods include evaporation, ion exchange, adsorption, flocculation, electro dialysis, solvent extraction, co-precipitation, and chelating therapy [6,7]. Clay mineral can adsorb notable quantities of a variety of heavy metals in geologic systems due to their large specific surface areas, reactive surface properties, and high cationic exchange capacities. Previous works show that Pb ions mainly form outer-sphere complexes on the permanently charged sites of montmorillonite (MM) [8]. The main disadvantage of MM clay is related to high water adsorption by clay and the infeasibility of separation of heavy metals. The surface properties of clay minerals can promote by replacing the exchangeable interlayer cations with organic cationic surfactants (e.g. intercalation) [9]. Normally, organic surfactants are used to make the surface of clay platelets organophilic and swell the clay galleries [9,10]. These modified organo-clays, which are used in a wide range of particular applications, such as adsorbents for organic pollutants metal ions and catalysts [11]. The suitability of organically modified NCs (NCs) as an adsorbent for the adsorption of organic and inorganic pollutants from industrial effluents can be due to their nano-size, specific surface area, and also their great tendency to absorb ions and organic compounds [10]. This research was conducted to optimize Pb^2+^ adsorption onto hexadecyl trimethyl ammonium chloride surfactant (N-HTAC) using the response surface methodology (RSM). The classical method, by changing one factor and fixing others, is not as precise and reliable to optimize of parameters, because it does not depict the interactive effects between all the factors involved, spend a lot of time and require numerous tests. These limitations can remove by using the RSM [[12], [13], [14]]. Therefore, in this work, the R software was applied for optimization and modelling the effects of multiple variables and their response, in which all factors are varied simultaneously [15]. In R software, RSM has a dual aim to find the optimum settings for the variables and to see how the variables perform over the whole experimental domain, including any interactions, and covers the most standard first- and second-order designs and methods for one response variable; but it covers those reasonably well [[11], [12], [13],^16^,^17^].

### Materials and methods

#### Preparation and characterization of N-HTAC

Hexadecyl trimethyl ammonium chloride (C_19_H_42_ClN, purity≥99% and molar weight = 320 g mol^−1^), was purchased from Merck Chemical Co. Nano-clay (Cloisite Na^+^) was bought from Gonzales Co (Texas, USA). The mineral type of nanoparticle was montmorillonite. Different concentrations of the HTAC surfactant were added to the Erlenmeyer flasks (250 mL). Then, the NC (3 g) was added to each of the flasks. The samples were stirred (one day) on the shaker (20^ºC^, 300 rpm, and pH 7.0) and then they were centrifuged. Materials obtained were washed five times with pure water and then were dried in an oven (at 105^°C^). Thus, the four types of modified NC adsorbent with different concentrations of HTAC were prepared. Finally, the final product (modified adsorbent) were kept inside sealed polyethylene bottle. The surface structure of NC, before and after the modification was analyzed by Field-Emission Scanning Electron Microscopy (FESEM) coupled with Energy Dispersive X-ray analysis (EDAX) using the Cambridge-Leo system [at 15 kV with background subtraction with a summation of 240 scans]. The scanning speed was 0.02 s^-1^. The d-spacing of the organic MM was analyzed using Bragg’s equation (nk = 2dsinh), Where n, k, h, and d are an integer, the wavelength, the glancing angle of incidence, and the interplanar spacing of the crystal, respectively. For measuring compound formation, the powder x-ray diffraction (PXRD) technique was used [with Cu Kα radiation source over a range of 10–120 at 1.54^Å^ wavelength, a scan speed 1 s step^-1^, and 25^°C^]. To accurately measure the total area of porous samples and calculation the distribution of pore size, the Brunauer-Emmett-Teller (BET) and Barrett- Joyner- Halenda (BJH) be employed, respectively.

#### Analysis and adsorption experiment

In this research, the Pb^2+^ cation was used as an adsorbate supplied by *Merck* Co. A stock solution of Pb^2+^ (1000 mg L^−1^) was prepared. Then, desired Pb^2+^ concentrations were obtained by the dilution of the stock solution (based on equation of C_1_V_1_=C_2_V_2_). The experiments were conducted in Erlenmeyer flasks (250 mL) so that 100 mL desired concentration of Pb^2+^ was added to the flasks in each run. The solution pH was adjusted to the desired amount and required dosages of adsorbent and surfactant were added to the Erlenmeyer flasks. The solutions were mixed for a specified time period. After the mixing, the samples were centrifuged to separate the adsorbent (5 min, 5000 rpm). After centrifuging, the residual Pb^2+^concentration was measured using ICP-OES

#### Factorial experimental design and optimization

Modelling and prediction relation between independent factors and one dependent response was done by the RSM technique using the central composite design (CCD). The R (programming language) software for Windows (version 3.0.3:6 March 2014) [13] was applied for the technique. The CCD procedure in R software is included factorial portion, star points, and several center points. Table 1 presents the independent variables used for experimental design. A full factor design (considering six independent variables and one dependent response) was carried out using a 2^6^ full factorial (2^n^), 12-star points, and 36 replicates in the center points [6,18,19]. For the data, the three RSM techniques [20,21] including full second-order model, first-order response surface model, and two-way interactions model were fitted [20,21]. To evaluate the accuracy of model fitting, the analysis of variance (ANOVA) by the good agreement of multiple R^2^ with adjusted R-squared (R_adj_) and insignificant lack of fit (LoF) was used [21]. To evaluate the selected model, also, the values of F_value_, P_value_, and LoF were checked. The model with the greater F_value_ and the smaller P_value_ and also insignificant LoF (or more LoF) was selected as a significant model [20,[22], [23], [24]]. A quadratic model as Eq. 1 was used to the interaction between (ϒ) and (independent variables):

(1) $$ϒ=b_{0}+\sum_{i=1}^{k}b_{i}X_{i}+\sum_{i=1}^{k}b_{ii}X_{i}^{2}+\sum_{i=1}^{k-1}\sum_{j=1}^{k}b_{ij}X_{i}X_{j}+C$$

Where, b_0_, b_i_, b_ii_, and b_ij_ are intercepted value, the regression coefficient for the linear, second-order, and interactive effects, respectively. X_i_, X_j_, and C are the independent variables, and C denotes the error of prediction, respectively. To obtain optimum conditions, finally, the Solver “Add-ins” was applied using effective parameters [21].

**Table 1:** Real and coded values of independent variables used for experimental design.

Variable	Symbol	Coded level		
		−1	0	1
		Real values		
pH	X_1_	3	6	9
Adsorbent dose (g L^−1^)	X_2_	0.5	2.75	5
Time (min)	X_3_	5	62.5	120
Initial arsenic concentration (mg L^−1^)	X_4_	0.1	0.55	1
Temperature	X_5_	10	22.5	35
HTAC surfactant dose (g L^−1^)	X_5_	0.5	2.75	5

### Results

**Fig. 1:**
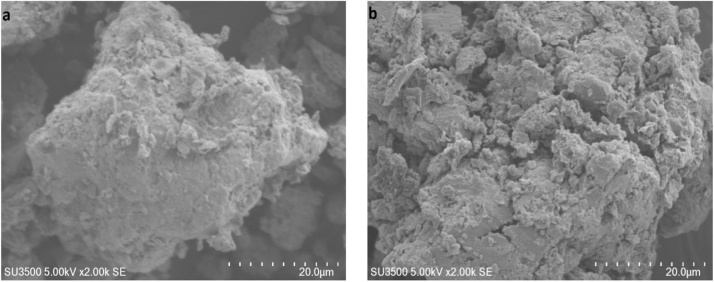
SEM images of NC (a) and N-HTAC (b).

**Fig. 2:**
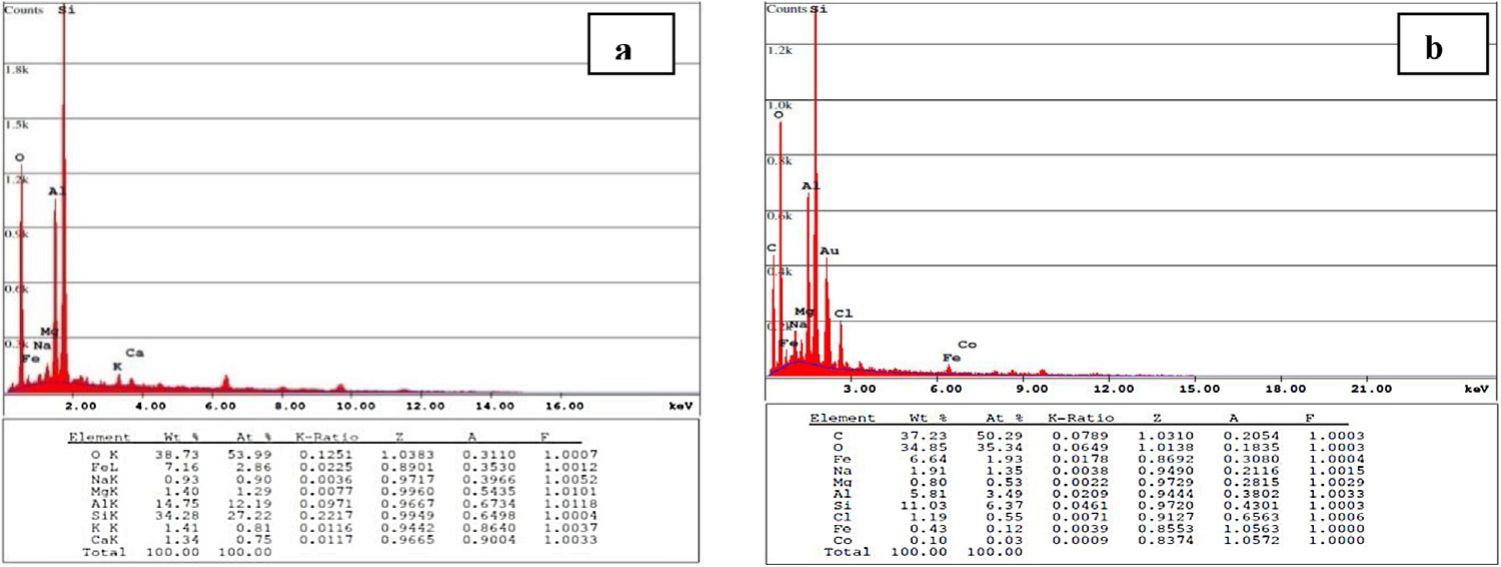
EDAX analysis for determination of element compounds contained in the NC, before (a) and after (b) modification (N-HTAC).

**Fig. 3:**
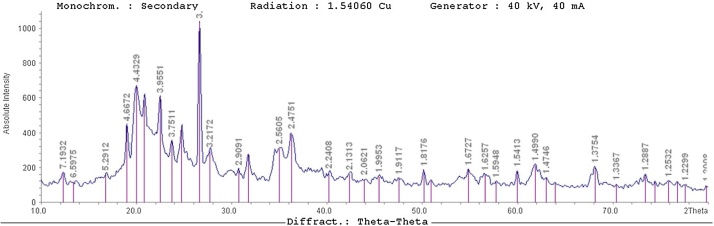
The XRD analysis for determination of the minerals in N-HTAC.

**Fig. 4:**
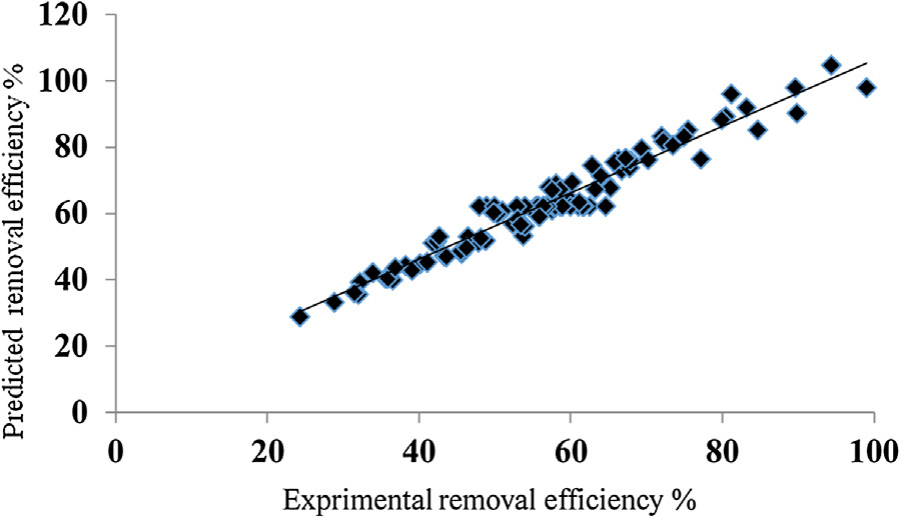
Experimental Pb^+2^ removal vs. predicted removal efficiency.

**Fig. 5:**
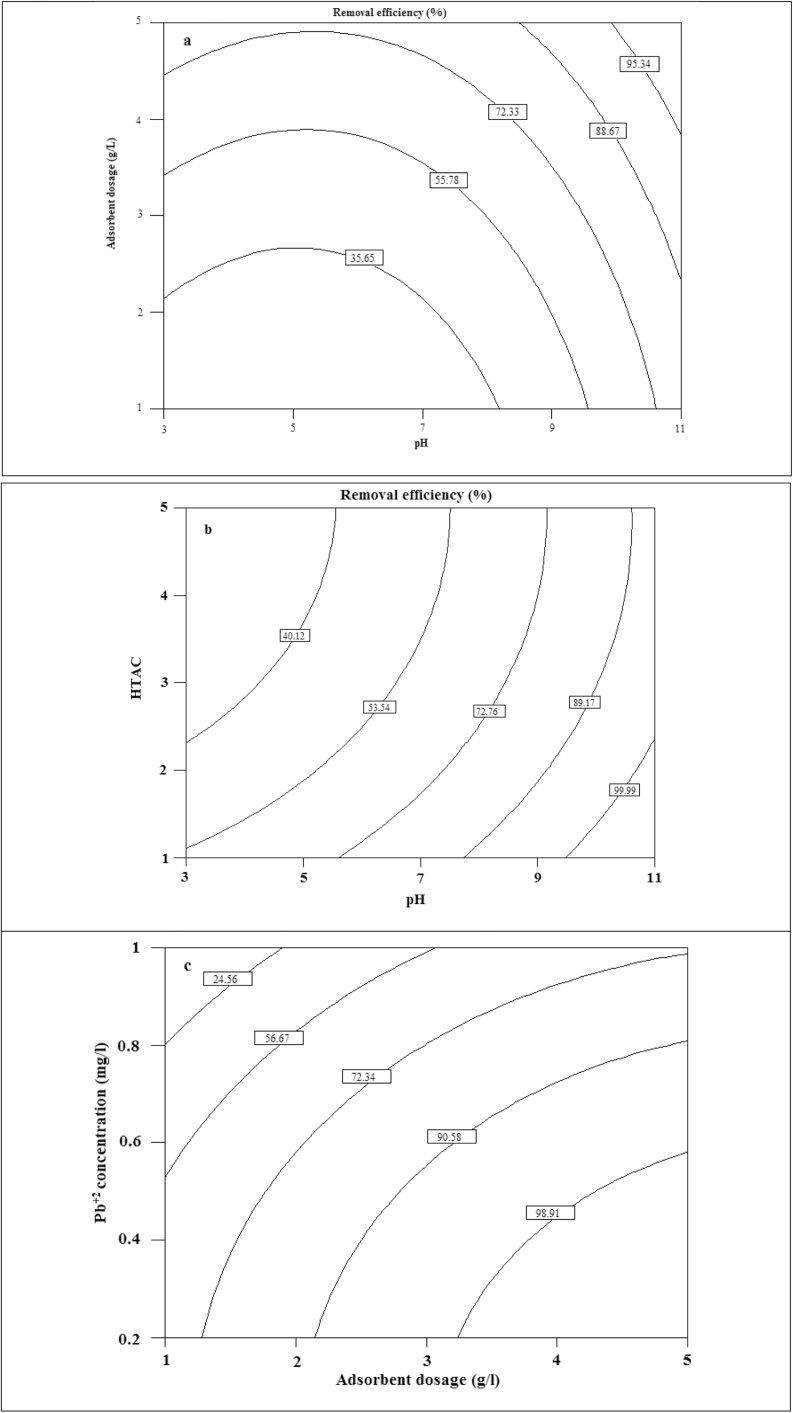
Contour plot for the effect of pH and adsorbent (a), pH and HTAC concentration (b), adsorbent dosage and initial Pb^+2^ concentration (c).

Fig. 1 exhibits the FE-SEM images of the NC (Fig.1-a) and NC modified with organic surfactant (Fig. 1-b). The results of EDAX analysis are presented in Fig. 2. Fig. 3 presents the XRD analysis. Table 2 shows the matrix of CCD with un-coded values of the independent variables and experimental and predicted values of the response. The obtained results from the comparison of the CCD technique are presented in Table 3. Table 4 presents the ANOVA analysis (for the reduced full second-order model). Table 5 shows the regression results of the reduced quadratic model (with coded and un-coded values of the independent variables). Experimental Pb^+2^ removal efficiency versus predicted removal efficiency is shown in Fig. 4. The effect of an interactive effect between two variables pH and adsorbent dose on Pb^2+^ adsorption is shown in Fig. 5-a. In the case of an interactive effect between pH and surfactant dosage, a similar trend was observed in Fig. 5-b and -c shows the interactive effect between adsorbent dose and initial concentration of adsorbate on the removal percentage of Pb^2+^.

**Table 2:** Central composite design matrix with un-coded values of the independent variables and experimental and predicted values of the response.

Sl.no.	Un-coded values						Removal	
	X_1_	X_2_	X_3_	X_4_	X_5_	X_6_	Expt. (ϒ)	Pred. (ϒ)
1	9	2.75	62.5	0.55	22.5	2.75	89.81	90.18
2	7.06	3.54	82.83	0.71	26.92	3.54	81.13	95.97
3	7.06	3.54	82.83	0.71	26.92	1.95	71.99	83.18
4	7.06	3.54	82.83	0.71	18.08	3.54	80.44	89.35
5	7.06	3.54	82.83	0.71	18.08	1.95	66.29	76.56
6	7.06	3.54	82.83	0.39	26.92	3.54	94.35	104.65
7	7.06	3.54	82.83	0.39	26.92	1.95	83.20	91.86
8	7.06	3.54	82.83	0.39	18.08	3.54	89.66	98.02
9	7.06	3.54	82.83	0.39	18.08	1.95	75.51	85.23
10	7.06	3.54	42.17	0.71	26.92	3.54	69.36	79.71
11	7.06	3.54	42.17	0.71	26.92	1.95	58.22	66.92
12	7.06	3.54	42.17	0.71	18.08	3.54	66.67	73.09
13	7.06	3.54	42.17	0.71	18.08	1.95	50.53	60.29
14	7.06	3.54	42.17	0.39	26.92	3.54	79.89	88.38
15	7.06	3.54	42.17	0.39	26.92	1.95	65.75	75.59
16	7.06	3.54	42.17	0.39	18.08	3.54	72.20	81.76
17	7.06	3.54	42.17	0.39	18.08	1.95	58.06	68.97
18	7.06	1.95	82.83	0.71	26.92	3.54	70.19	76.32
19	7.06	1.95	82.83	0.71	26.92	1.95	60.05	63.53
20	7.06	1.95	82.83	0.71	18.08	3.54	64.51	69.69
21	7.06	1.95	82.83	0.71	18.08	1.95	52.36	56.90
22	7.06	1.95	82.83	0.39	26.92	3.54	73.41	80.56
23	7.06	1.95	82.83	0.39	26.92	1.95	65.27	67.76
24	7.06	1.95	82.83	0.39	18.08	3.54	67.72	73.93
25	7.06	1.95	82.83	0.39	18.08	1.95	57.58	61.14
26	7.06	1.95	42.17	0.71	26.92	3.54	55.43	60.05
27	7.06	1.95	42.17	0.71	26.92	1.95	43.28	47.26
28	7.06	1.95	42.17	0.71	18.08	3.54	53.74	53.43
29	7.06	1.95	42.17	0.71	18.08	1.95	35.59	40.64
30	7.06	1.95	42.17	0.39	26.92	3.54	59.96	64.29
31	7.06	1.95	42.17	0.39	26.92	1.95	47.81	51.50
32	7.06	1.95	42.17	0.39	18.08	3.54	53.27	57.67
33	7.06	1.95	42.17	0.39	18.08	1.95	40.12	44.88
34	6	5	62.5	0.55	22.5	2.75	99.03	98.07
35	6	2.75	120	0.55	22.5	2.75	84.65	85.29
36	6	2.75	62.5	1	22.5	2.75	46.49	53.16
37	6	2.75	62.5	0.55	35	2.75	63.24	67.43
38	6	2.75	62.5	0.55	22.5	5	77.19	76.45
39	6	2.75	62.5	0.55	22.5	2.75	57.78	62.29
40	6	2.75	62.5	0.55	22.5	2.75	62.43	62.29
41	6	2.75	62.5	0.55	22.5	2.75	58.61	62.29
42	6	2.75	62.5	0.55	22.5	2.75	56.78	62.29
43	6	2.75	62.5	0.55	22.5	2.75	61.61	62.29
44	6	2.75	62.5	0.55	22.5	2.75	57.61	62.29
45	6	2.75	62.5	0.55	22.5	2.75	55.78	62.29
46	6	2.75	62.5	0.55	22.5	2.75	55.43	62.29
47	6	2.75	62.5	0.55	22.5	2.75	64.61	62.29
48	6	2.75	62.5	0.55	22.5	2.75	55.61	62.29
49	6	2.75	62.5	0.55	22.5	2.75	57.78	62.29
50	6	2.75	62.5	0.55	22.5	2.75	56.93	62.29
51	6	2.75	62.5	0.55	22.5	2.75	61.73	62.29
52	6	2.75	62.5	0.55	22.5	2.75	56.43	62.29
53	6	2.75	62.5	0.55	22.5	2.75	61.03	62.29
54	6	2.75	62.5	0.55	22.5	2.75	57.93	62.29
55	6	2.75	62.5	0.55	22.5	2.75	61.53	62.29
56	6	2.75	62.5	0.55	22.5	2.75	59.93	62.29
57	6	2.75	62.5	0.55	22.5	2.75	52.93	62.29
58	6	2.75	62.5	0.55	22.5	2.75	57.93	62.29
59	6	2.75	62.5	0.55	22.5	2.75	58.93	62.29
60	6	2.75	62.5	0.55	22.5	2.75	48.93	62.29
61	6	2.75	62.5	0.55	22.5	2.75	47.93	62.29
62	6	2.75	62.5	0.55	22.5	2.75	47.93	62.29
63	6	2.75	62.5	0.55	22.5	2.75	53.93	62.29
64	6	2.75	62.5	0.55	22.5	2.75	56.93	62.29
65	6	2.75	62.5	0.55	22.5	2.75	56.93	62.29
66	6	2.75	62.5	0.55	22.5	2.75	47.93	62.29
67	6	2.75	62.5	0.55	22.5	2.75	58.93	62.29
68	6	2.75	62.5	0.55	22.5	2.75	56.93	62.29
69	6	2.75	62.5	0.55	22.5	2.75	56.93	62.29
70	6	2.75	62.5	0.55	22.5	2.75	56.38	62.29
71	6	2.75	62.5	0.55	22.5	2.75	56.38	62.29
72	6	2.75	62.5	0.55	22.5	2.75	53.93	62.29
73	6	2.75	62.5	0.55	22.5	2.75	49.93	62.29
74	6	2.75	62.5	0.55	22.5	2.75	52.93	62.29
75	6	2.75	62.5	0.55	22.5	0.5	45.58	48.14
76	6	2.75	62.5	0.55	10	2.75	45.50	48.69
77	6	2.75	62.5	0.1	22.5	2.75	63.97	71.42
78	6	2.75	5	0.55	22.5	2.75	32.18	39.29
79	6	0.5	62.5	0.55	22.5	2.75	33.88	42.21
80	4.94	3.54	82.83	0.71	26.92	3.54	62.76	74.66
81	4.94	3.54	82.83	0.71	26.92	1.95	58.63	67.43
82	4.94	3.54	82.83	0.71	18.08	3.54	57.067	68.03
83	4.94	3.54	82.83	0.71	18.08	1.95	50.94	60.81
84	4.94	3.54	82.83	0.39	26.92	3.54	74.97	83.33
85	4.94	3.54	82.83	0.39	26.92	1.95	67.84	76.11
86	4.94	3.54	82.83	0.39	18.08	3.54	67.28	76.71
87	4.94	3.54	82.83	0.39	18.08	1.95	60.15	69.48
88	4.94	3.54	42.17	0.71	26.92	3.54	52.99	58.39
89	4.94	3.54	42.17	0.71	26.92	1.95	41.86	51.17
90	4.94	3.54	42.17	0.71	18.08	3.54	42.29	51.77
91	4.94	3.54	42.17	0.71	18.08	1.95	38.17	44.55
92	4.94	3.54	42.17	0.39	26.92	3.54	57.52	67.07
93	4.94	3.54	42.17	0.39	26.92	1.95	50.39	59.84
94	4.94	3.54	42.17	0.39	18.08	3.54	49.83	60.44
95	4.94	3.54	42.17	0.39	18.08	1.95	42.69	53.22
96	4.94	1.95	82.83	0.71	26.92	3.54	55.87	59.25
97	4.94	1.95	82.83	0.71	26.92	1.95	48.74	52.03
98	4.94	1.95	82.83	0.71	18.08	3.54	48.18	52.63
99	4.94	1.95	82.83	0.71	18.08	1.95	41.05	45.41
100	4.94	1.95	82.83	0.39	26.92	3.54	61.09	63.49
101	4.94	1.95	82.83	0.39	26.92	1.95	53.96	56.27
102	4.94	1.95	82.83	0.39	18.08	3.54	53.39	56.87
103	4.94	1.95	82.83	0.39	18.08	1.95	46.27	49.64
104	4.94	1.95	42.17	0.71	26.92	3.54	39.10	42.99
105	4.94	1.95	42.17	0.71	26.92	1.95	31.97	35.77
106	4.94	1.95	42.17	0.71	18.08	3.54	31.41	36.36
107	4.94	1.95	42.17	0.71	18.08	1.95	24.28	29.14
108	4.94	1.95	42.17	0.39	26.92	3.54	43.63	47.23
109	4.94	1.95	42.17	0.39	26.92	1.95	36.50	40.00
110	4.94	1.95	42.17	0.39	18.08	3.54	35.94	40.60
111	4.94	1.95	42.17	0.39	18.08	1.95	28.81	33.38
112	3	2.75	62.5	0.55	22.5	2.75	36.80	43.77

**Table 3:** The comparison of different models of RSM for fitting a response-surface model.

	Multiple R-squared	Adjusted R-squared	F-statistic	P_value_	AIC	LoF
First-order response-surface model	0.922	0.917	207.6 on 6 and 105 DF	< 2.2E-16	110.57	0.6829
Two-way interactions model	0.938	0.923	64.84 on 21 and 90 DF	< 2.2E-16	124.14	0.8186
Second-order model	0.949	0.933	58.73 on 27 and 84 DF	< 2.2E-16	38.74	0.9696
Reduced full second-order model	0.945	0.936	110.2 on 15 and 96 DF	< 2.2E-16	21	0.9696

**Table 4:** Analysis of variance (ANOVA) for the reduced full second-order model.

Model formula in RSM	DF	Sum of squares	mean square	F-value	probability (P)
First-order response (x_1_, x_2_, x_3_, x_4_, x_5,_ x_6_)	6	19619.1	3269.9	268.82	<2.2E-16
Two-way interaction response (x_1,_ x_2,_ x_4_, x_6_)	6	275.5	45.9	3.77	0.002037
Pure quadratic response (x_1_, x_2_, x_5_)	3	210.6	70.2	5.77	0.001135
Residuals	96	1167.7	12.2	–	–
LoF	61	567.9	9.3	0.54	0.981816
Pure error	35	599.8	17.1	–	–

*Notes*: multiple R-squared = 0.945, Adjusted R-squared = 0.936, Predicted R-squared = 0.923, F-statistic: 110.2 on 15 and 96 DF, P_value_: <2.2E-16.

**Table 5:** Regression analysis of the reduced model with coded and un-coded values of the independent variables.

Model term	Coded values				Un-coded values			
	Coefficient estimate	Std. error	t-value	p-value	Coefficient estimate	Std. error	t-value	p-value
(Intercept)	56.12935	0.48364	116.0555	<2.2E-16	−14.74760	17.98921	−0.820	0.41430
X_1_	23.25837	1.10288	21.0889	<2.2E-16	−6.50814	3.74878	−1.736	0.08566
X_2_	22.71207	1.10288	20.5935	<2.2E-16	−1.14511	4.43636	−0.258	0.79685
X_3_	23.08574	1.10288	20.9323	<2.2E-16	0.40149	0.01889	21.252	<2E-16
X_4_	−9.13412	1.10288	−8.2821	7.161E-13	3.80691	9.63727	0.395	0.69368
X_5_	9.37827	1.10287	8.5035	2.417E-13	1.96873	0.68873	2.859	0.00519
X_6_	1.41700	0.11029	12.8483	<2.2E-16	−3.61332	3.09140	−1.169	0.24528
X_1_:X_2_	8.47500	3.48760	2.4300	0.016955	1.25556	0.50891	2.467	0.01534
X_1_:X_6_	1.11500	0.34876	3.1970	0.001880	1.65185	0.50891	3.246	0.00160
X _2_:X _4_	−8.87500	3.48760	−2.5447	0.012530	−8.76543	3.39274	−2.584	0.01124
X_1_^2^	−20.30208	2.40852	1.9511	0.053965	0.52213 1.55037	0.26359	1.981	0.05038
X_2_^2^	7.84874	2.40852	3.2587	0.001548	−0.02708	0.46860	3.308	0.00131
X_5_^2^	−4.23080	2.40852	−1.7566	0.082175	0.52213 1.55037	0.01518	−1.783	0.07758

### Discussion

#### Characterization of the sorbent

The morphology of NC was ascertained with the aid of FE-SEM. Fig. 1 exhibits the FE-SEM images of the NC (Fig. 1-a) and NC modified with organic surfactant (Fig. 1-b). As seen in Fig. 1, this NC is a porous structure with a vast surface which is indeed responsible for its good adsorbing capacity. The results of EDAX analysis are presented in Fig. 2. The presence of a carbon atom in the modified NC represents to create organic NC. Furthermore, it was found that after the modification, the amounts of all elements other than sodium were decreased. Moreover, inorganic NC (N-HTAC), iron (Fe) and cobalt (Co) were replaced with potassium (K^+^) and calcium (Ca^2+^), respectively. Fig. 3 presents the XRD analysis. Actually, Fig. 3 describes organic NC minerals, which include clay (Askmtyt and Kandyt), carbonate (calcite), silicate (Quartz), and philo silicate (Kaolinite) and sub-groups (Illite, Muscovite, Poligourcite, and Calcite). In some analyzed samples, also, Gypsum has been observed. Based on the results of BET-BJH analysis, the smallest diameter, the average particle diameter and surface area to volume for NC were 1.66 nm, 6.1 nm, and 2.7 m^2^ g^−1^, respectively. Also, the smallest diameter, the average particle diameter, and surface area to volume for N-HTAC were 1.66 nm, 6.1 nm, and 2.7 m^2^ g^−1^, respectively.

#### Adsorption modelling

Table 2 shows the CCD matrix with un-coded values of the independent variables and experimental and predicted values of the response. Based on CCD, it generated 112 runs by 2^6^ full factorial, 12-star points, and 36 replicates in the center points (Table 2). This method has applied for specification the response-surface portion of the model. The ANOVA is used as a statistical technique to depict model adequacy, [19,23]. The ANOVA introduced information about P_value_, F_value_, multiple R^2^, adjusted R^2^, AIC, and LoF. The LoF determines data variation around the fitted model and must be insignificant in a well-fitted model for each model to appraise model adequacy. The model with (i) the smaller P_value_ and AIC (ii) the higher F_value_, multiple R^2^, adjusted R^2^ and also (iii) the insignificant LoF, was selected as a suitable model for Pb^2+^ adsorption on N-HTAC [25]. The results obtained from the comparison of the CCD presents in Table 3. It observed a breakdown or LoF with small P_value_ 0.6829 and <2.2E-16 for the first-order response-surface and two-way interactions models, respectively. The results also revealed the lower multiple R^2^, adjusted R^2^ and the higher AIC for these models in comparison with ones for the full second-order model. It also achieved information about the stationary point of response surface but in the first-order response-surface and two-way interactions models due to a significant LoF for them, the stationary point in original unit information is of little use (data are not shown) [21,25]. Therefore, we are tried to apply to a full second-order model because of obtaining the higher multiple R^2^ (0.949), the adjusted R^2^ (0.933) and F_value_ (58.73 on 27 and 84 DF)), the lower AIC (38.74) and (insignificant LoF (0.969) than previous models. It also observed the R^2^ value of the selected model was very close to the adjusted R^2^, representing satisfactory adjustment between full second-order model and experimental data [21,26]. To the development of regression model equation, the reduced full second-order model generated with removing some insignificant items from the full second-order model. Table 4 present the ANOVA analysis (for the reduced full second-order model). Table 5 shows the regression results of the reduced quadratic model (with coded and un-coded values of the independent variables). Based on Table 5, six selected independent variables, the interaction effect and also pure quadratic response for x_1,_ x_2_, and x_5_ have significant effect on Pb^+2^ adsorption. Therefore, these terms could be an impressed model. Also, the predicted equation (e.g. Final equation) by the model for coded (Eq. 2) and un-coded (Eq. 3) values of the independent variables are presented as follows:

(2) ϒ=56.13+23.25X_1_+22.71X_2_+23.08X_3_-9.13X_4_+9.37X_5_+1.41X_6_+4.69X_1_^2^+7.84X_2_^2^-4.23X_5_^2^+8.47X_1_X_2_+1.11X_1_X_6_-8.87X_2_X_4_

(3) ϒ=-14.74-6.50X_1_-1.14X_2_+0.40X_3_+3.80X_4_+1.96X_5_-3.61X_6_+0.52X_1_^2^-1.55X_2_^2^-0.027X_5_^2^+1.25X_1_X_2_+1.65X_1_X_6_-8.76X_2_X_4_

As presented equations, it obvious that x_1_, x_2_, x_3_, x_4_, x_5_, x_6_, x_1_:x_2_, x_1_:x_6_, x_1_^2^, and x_2_^2^ terms have a synergistic effect on the response prediction by the model while x_2_:x_4_ and x_5^_^2^ terms show the antagonistic effect on the model. Experimental Pb^+2^ removal efficiency versus predicted removal efficiency is shown in Fig. 4. It was observed a good agreement between the experimental data value and the values predicted by the model. Therefore predicted model can be used for prediction and optimization [27,28].

#### Validation modeling

The summary of the reduced full second-order model indicated information about the stationary point in original units. This information is too close to the experimental region and it is experimentation-clear evidence of a nearby set of optimal condition. Therefore, it should probably collect some confirmatory data near this estimated optimum to make sure (data are not shown). The Solver “Add-ins” software was applied to confirm and to obtain optimum conditions for model predicted by RSM [21]. The optimum operating points giving maximum Pb^2+^ removal (99.99%) and to involve all parameters simultaneously, was determined: initial Pb^2+^ concentration, 0.1 mg L^−1^; adsorbent dosage, 4.33 g L^−1^; surfactant dosage, 4.19 g L^−1^; pH, 7.13; temperature, 28.06^ºC^, and the contact time, 103.4 min. The results were too close to the stationary point in original units. To confirm the validity of the predicted optimum conditions, an additional experiment was carried out. The results indicated that experimental findings for response were in good agreement with the model prediction [20].

#### Investigating the effect of main variables on Pb^2+^ adsorption

Interaction effects of the dependent variables on the removal Pb^2+^ efficiency were expressed by the contour plotting. Solution pH is one of the most important parameters which reflects the adsorption capacity of adsorbent [[29], [30], [31]]. It effects the ionization of functional groups on the adsorbent surface and charges of adsorption sites. Thus, the effects of solution pH were studied in the range of 3 to 9. The effect of an interactive effect between two variables of pH and adsorbent dose is shown in Fig. 5-a. According to Fig. 5-a, the removal efficiency of Pb^2+^ was increased with increasing in adsorbent dosage. This can be attributed to more available sites, the diffusion of the Pb^2+^ into the bulk of the adsorbent and increasing in the number of linking sites of active and accessible for the sorbent [32,33]. It was observed an increase in the quantitative removal of Pb^2+^ with increasing pH from pH 3 to 9. A lower removal efficiency under highly acidic conditions can be due to the occupation of active sites of N-HTAC by H^+^ and H_3_O^+^. Under these conditions, the surface of adsorbent was protonated and was positively charged resulting in a repulsive force between adsorbate and adsorbent and excessive H^+^ fought with Pb^2+^ in the solution for an ion exchange reaction with unsaturated ions in N-HTAC [29,34]. The pH_zpc_ (pH_zero point charge_) of adsorbent is very important in the determination of optimum pH [31,35]. In this pH, the surface charge is neutral and at pH levels lower and higher than the pH_zpc_, surface charge is negative and positive, respectively [23,24]. In pH with a negative charge (higher than pH_zpc_), the cations of Pb^2+^ can interact electrostatically with the N-HTAC resulting promote the quantitative removal, while in pH levels with a positive charge (lower than pH_zpc_) does not favor the pb^2+^adsorption due to the electrostatic repulsion. In this study, the pH_zpc_ of adsorbent was determined at 6.5. In the case of an interactive effect between pH and surfactant dosage, a similar trend was observed in Fig. 5-b. According to Fig. 5-b, an increase in the surfactant dosage (0.5–5 g L^−1^) resulted in an increase in the response percentage. The phenomenon can be due to the presence of an organic medium created in the NC resulting in large molecular size and also the high capacity of the NC for enlarging its interlayer space. The BET analysis proved this characteristic, as the surface area for the NC and the N-HTAC was obtained 4.46 and 61.99 m^2^  g^−1^NC, respectively. Also, the BJH analysis revealed a mean pore diameter of 6.1 nm for NC in comparison with 7.5 nm for N-HTAC. Thus, these results expained a higher removal efficiency of pb^2+^ by N-HTAC. Fig. 5-c shows the interactive effect between adsorbent dose and initial concentration of adsorbate on the removal efficiency of Pb^2+^. Based on Fig. 5-c, it found that the removal efficiency was decreased with an increase in the initial Pb^+2^ concentration. This can be due to the saturation of binding sites with increasing concentration because, for a given mass of adsorbent, the surface binding sites on the adsorbent are fixed resulting decrease in removal efficiency. It expresses that the internal part of the adsorbent has a low role in Pb^2+^ sorption, and the main adsorption of Pb^2+^ is related to the adsorbent surface. In Fig. 5-c, a similar trend for adsorbent dose was observed (as Fig. 5-b).

### Conclusions

To increase Pb^2+^ adsorption, in present study, NC (Montmorillonite) modified by hexadecyl trimethyl ammonium chloride was used. The study was performed in batch conditions. To investigate the relationship between input independent variables and one dependent output response, the response surface methodology using a central composite design was used. The reduced full second-order model was applied for prediction and optimization of data using Solver “Add-ins” in Microsoft Excel 2010. Results indicated that the reduced full second-order model has highly significant on Pb^2+^ adsorption onto N-HTAC with the P_value_ (<2.2E-16), R^2^ (multiple R-squared: 0.945, adjusted R-squared: 0.936), insignificant LoF (0.98) and AIC. It observed satisfactory agreement between model and experimental data. It was obvious that x_1_, x_2_, x_3_, x_4_, x_5_, x_6_, x_1_:x_2_, x_1_:x_6_, x_1_^2^, and x_2_^2^ terms have a synergistic effect on the response prediction by the model while x_2_:x_4_ and x_5_^2^ terms show the antagonistic effect on the model. All these terms entered into the model due to P_value_<0.05. The optimum operating points giving maximum Pb^+2^ removal (99.99%) and to involve all parameters simultaneously, was determined: initial Pb^+2^ concentration, 0.1 mg L^−1^; adsorbent dosage, 4.33 g L^−1^; surfactant dosage, 4.19 g L^−1^; pH, 7.13; temperature, 28.06^ºC^ and the contact time, 103.4 min.
